# The prevalence of post traumatic and associated factors among nurses during COVID_19 pandemics: a systematic review and meta-analysis

**DOI:** 10.1186/s40359-024-01578-z

**Published:** 2024-05-16

**Authors:** Afsaneh Karbasi, Masoudeh Babakhanian, Akram Ahmadzadeh, Victoria Omranifard

**Affiliations:** 1 Department of Psychiatry, Child and Adolescent Psychiatry, Isfahan University of Medical Sciences, Isfahan, Iran; 2 Social Determinants of Health Research Center, Semnan University of Medical Sciences, Semnan, Iran; 3 Department of Counselling, University of Social Welfare and Rehabilitation Sciences, Tehran, Iran; 4 Department of Psychiatry, Behavioral Sciences Research Center, Isfahan University of Medical Sciences, Isfahan, Iran

**Keywords:** COVID-19, Meta-analysis, Nurse, Post-traumatic growth, Prevalence

## Abstract

**Background:**

Despite the negative outcomes, exposure to a crisis may cause people to experience positive changes. This study aims to analyze the prevalence of post-traumatic growth (PTG) and its relevant factors among nurses during the COVID-19 pandemic.

**Method:**

The research protocol was registered with PROSPERO (CRD42022329671), and PRISMA steps were taken in this study. PubMed, Scopus and ProQuest were explored on 1/9/2022 to create the research database. According to the inclusion criterion, all studies analyzing the prevalence of post-traumatic growth through the PTG Inventory were considered eligible. They were all qualitatively assessed through the modified version of the Quality Assessment Checklist for prevalence studies.

**Results:**

A total of 15 papers met the inclusion criterion (*n* = 22756). According to the research results, the prevalence of PTG was randomly calculated ES [95% Conf. Interval = 0.15 [0.12–0.17]), and heterogeneity was reported I2 = 98.52% (*P* = 0.000). The results also indicated that the mean score of PTGI decreased in nurses as their work experience and mean age increased. However, the effect was not statistically significant for the mean age (*P* = 0.06). According to the results, the PTGI score decreased in nurses with more work experience, a finding which was statistically significant (*P* = 0.04).

**Conclusion:**

This meta-analysis determined a 15% prevalence rate of PTG in nurses. Psychological interventions should be developed and applied to older nurses with more work experience in order to mitigate the harm caused by the pandemic and its consequent crises.

## Background

The spread of a pandemic can often lead to a series of psychological problems in addition to physical complications [1]. Although such pandemics impact different parts of society, some people are more vulnerable than others due to their greater exposure to crises [2]. Nurses are at the frontier in this realm. In fact, since the outbreak of the COVID-19 pandemic, nursing services and relevant problems have frequently been discussed worldwide. The nurses involved in the COVID-19 cases are very susceptible to adverse psychological problems [3]. Due to exposure to traumatic situations, these nurses may experience negative psychological outcomes such as post-traumatic stress disorder (PTSD) [4], sleep problems [5], burnout [6], exhaustion [7], hopelessness [8], anger and depression [9].

However, not all nurses who experience and encounter a pandemic show such maladaptive responses. In addition to these negative outcomes, work-related traumatic events may lead to positive changes in nurses, something which is known as post-traumatic growth [10, 11].

Although, experiencing a traumatic event can shatter key elements of a person's worldview, beliefs, and goals and create a high level of psychological distress [12], but the study of the texts of different religions, the works of ancient philosophers, scientists of other fields, and also new researches show that the pain and suffering caused by negative experiences can lead to positive changes in different people and societies [13].

Proposed by Tedeschi and Calhoun, post-traumatic growth (PTG) is a concept defined as “positive psychological changes experienced as a result of coping with extremely challenging circumstances in life” [14, 15]. As Tedeschi and Calhoun have shown in their theoretical model of growth, what is important and significant in facing traumatic events and leads to behavioral, emotional, and cognitive reactions in people is the feeling of threat and danger [16]. Crises can not only threaten a person's life, but they can also shake a person's imaginary world and destroy a person's basic beliefs. As a result of both situations, the person experiences high emotional distress [17]. According to Tedeschi and Calhoun's model, the way to manage the emotional distress caused by the trauma experience is one of the predictors of growth or post-traumatic stress disorder [13]. Post-traumatic growth (PTG) is characterized by five areas: (1) increased appreciation for life, (2) more meaningful relationships, (3) increased sense of personal strength, (4) identifying new priorities, and (5) a richer existential and spiritual life [18]. According to some studies, PTG can improve the quality of life and boost the psychological state in people who experience traumatic events [17, 19]. During the COVID-19 pandemic, Kristine Olson and Martin Huecker emphasized the importance of investigating PTG and its facilitators among nurses [20, 21].

To address this stark knowledge gap, this systematic review aimed to analyze the predictors and perceived facilitators of PTG in nurses within quantitative, qualitative, and mixed-methods studies. Since the prevalence of PTG has not yet been systematically analyzed in nurses, this study aimed to investigate the problem and identify its effective factors.

## Materials and methods

The protocol of this study was registered in PROSPERO under the registration ID CRD42022329671.

### Search strategy

A systematic search on studies related to The Prevalence of post traumatic and associated factors among nurses during COVID_19 pandemics, was conducted on PubMed, Scopus and ProQuest databases. In addition, the list of review studies on the topic was manually searched to cover all related published articles. The following method was developed using a selection Medical Subject Headings (MeSH) from PubMed:

(COVID-19[mesh] OR COVID-19[tiab] OR “COVID 19”[tiab] OR COVID19[tiab] OR Coronavirus[tiab] OR SARS-CoV-2[tiab] OR “SARS CoV 2”[tiab] OR 2019-nCoV[tiab] OR “2019 nCoV Disease”[tiab]) AND (“Posttraumatic Growth, Psychological”[mesh] OR “Psychological Posttraumatic Growth”[tiab] OR Post-traumatic Growth, Psychological[tiab] OR Growth, Psychological Post-traumatic[tiab] OR Psychological Post-traumatic Growth[tiab] OR Psychological Post-traumatic Growth^*^[tiab] OR Posttraumatic Growth[tiab] OR Growth, Posttraumatic[tiab]) AND (nurse*[mesh]) 2020/01/01:2022/03/05[dp].

**Fig. 1:**
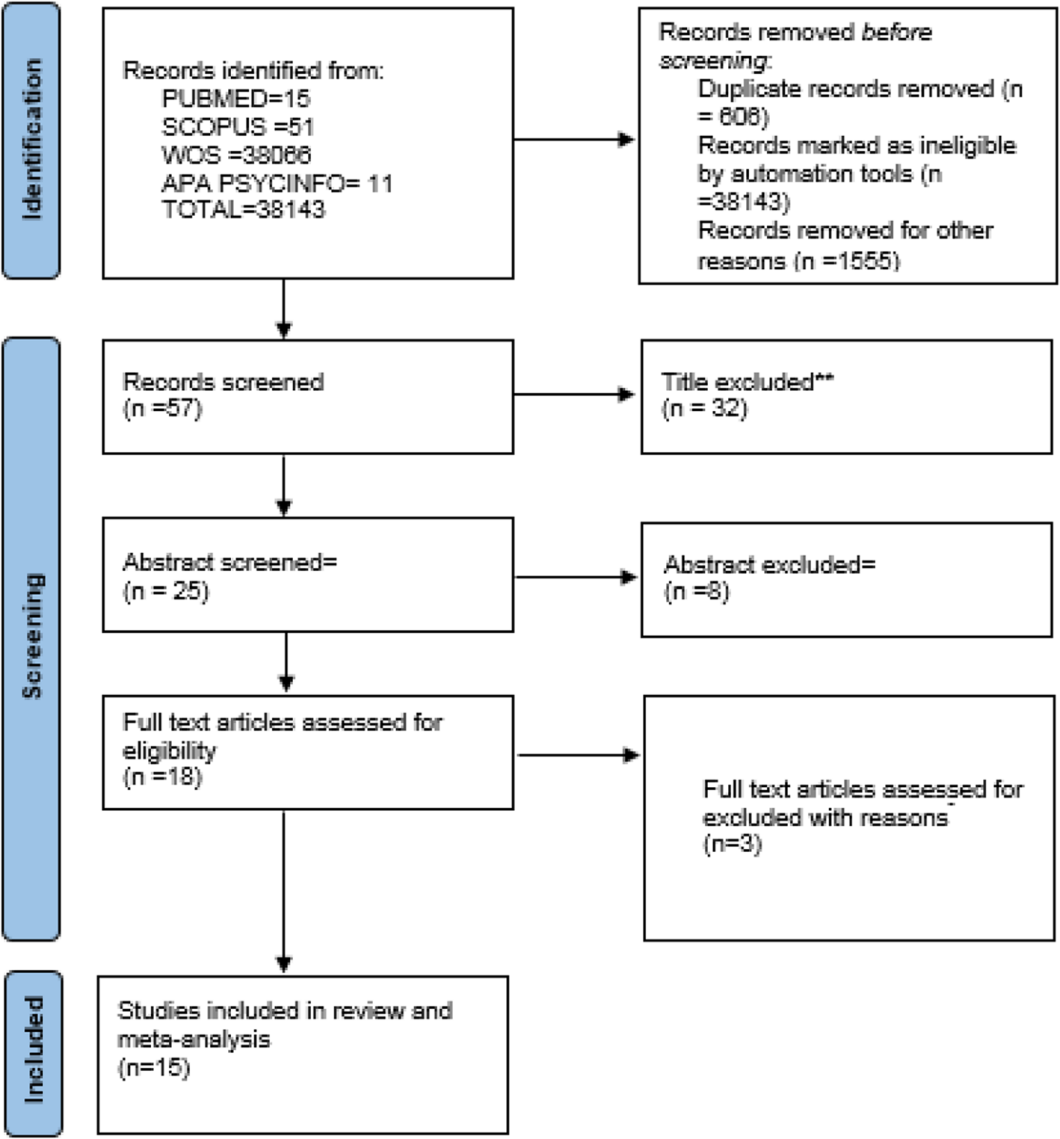
Identification of studies from databases and records based on the PRISMA flow diagram [36]

The search resulted in 101663 potential related articles on PubMed, Scopus and ProQuest databases. Next, 57 studies remained for full-text screening after removing duplicate records and title and abstract screening. In total, 42 studies were excluded because of unrelated topic, unsuitable design, and inaccessible full-text version. Finally, 15 studies entered in review and meta-analysis process [10, 22–35]. Figure 1 shows the flow diagram of the process of screening and selection.

A search strategy was adopted for each electronic database. POLIS (patients, outcome, location, indicator, and study design) was used to select the studies (Table 1). It is one of the most useful models to formulate observational studies regarding evidence combination, ensuring that the question’s components are well-defined.

**Table 1:** The POLIS (patients, outcome, location, indicator and study design)

POLIS criteria	Patients	Outcome	Location	Indicator	Study design
Description	Nurses in covid pandemic period	PTGI	All world	Prevalence of PTGI	Observational study with Cross-sectional, design

### Inclusion and exclusion criteria

The inclusion criterion was all studies examining prevalence of PTG using Post-traumatic Growth Inventory (PTGI).All studies in the selection steps for systematic review and meta-analysis are consisted of three steps namely title, abstract and full text. No limitations based on participant age, sex, ethnicity, language, race, journal language, or publication year were set for inclusion. Studies published in non-English languages were included if they could be translated to English easily using a web-based translation tool. Otherwise, they were excluded from the study. Cross-sectional observational studies were also included. Case studies (case reports or case series), studies with inaccessible full-text, and studies on other target groups were excluded.

### Outcome

The main desired outcome of this study was examining the prevalence of PTG in nurses working in hospitals during the COVID-19 pandemic.

### Selection of studies and extraction of data

The studies were selected by two independent authors (AA and MB) in all stages including screening, full-text examination, and qualitative assessment. Differences in views in any stage were resolved by consulting with a third independent examiner. Data extraction and qualitative assessment forms, designed in Microsoft Excel in advance, were given to the examiners. The variables were the name of the first author, publication year, study location, design type, sample size, mean age, target audience, work experience, instrument, mean score of developing PTG, PTG assessment outcome, and qualitative assessment score.

### Qualitative assessment of studies

Two examiners independently used the modified version of the quality assessment checklist for prevalence studies (adapted from Hoy et al.) for cross-sectional prevalence studies. It is a 10-item tool designed for assessing the risk of bias.

### Data synthesis

The research data on the prevalence of PTG in nurses were used to perform a meta-analysis by the metaprop command in STATA 12. The results were reported with 95% confidence interval. The I-squared test was used to identify heterogeneity. I^2^ < 25%, no heterogeneity; I^2^ = 25–50%, medium heterogeneity; I^2^ > 50%, high heterogeneity [37]. Funnel plot, trim and fill, and Egger’s test were used to assess publication bias. A p-value below 0.05 was considered statistically significant.

## Results

### Included studies

Table 2 shows the features of 15 articles selected from the total of systematically reviewed articles.

**Table 2:** Features of included studies

Authour, year	Place	Design	Sample sze	Male	Female	Age	Population	Instrument	Job experience	Results
Xin Tong Zhang, 2021 [22]	China	cross sectional study	1790	11	1779	≤ 30: 809 (45.20), 31 ~ 40: 675 (37.70), ≥ 41: 306 (17.10)	nurse	Post-Traumatic Growth Inventory (PTGI), Post-Traumatic Stress Disorder CheckList-Civilian Version (PCL-C)	≤ 5: 443 (24.70) 6 ~ 10: 628 (35.10) ≥ 11: 719 (40.20)	The analysis revealed that good social support and self-efficacy were important factors to improve the level of PTG of clinical nurses, while bad psychological state and working for many years were the negative factors of PTG
Pan pan Cui, 2020 [10]	China	cross sectional study	179	12	155	≤ 30: 104 (62.3), > 30: 63 (37.7)	nurse	Post-traumatic Growth Inventory (PTGI), Event-Related Rumination Inventory (ERRI)	≤ 3: 63 (37.7), 4 ~ 5 17: (10.2), 6 ~ 10: 47 (28.1), > 10 40 (24.0)	A total of 179 frontline nurses were recruited, and 167 were included in the analyses. The mean PTG score was 70.53 ± 17.26. The bivariate analyses showed that deliberate rumination was modestly positively correlated with PTG (*r* = 0.557, *p* < 0.01), while intrusive rumination had a modest negative correlation with PTG (*r* = − 0.413, *p* < 0.01). Multiple linear regression demonstrated that working years, self-confidence in frontline work, awareness of risk, psychological intervention or training during the epidemic and deliberate rumination were the main influencing factors of PTG among frontline nurses and accounted for 42.5% of the variance (F = 31.626, *p* < 0.001)
Xiaoxin Liu, 2021 [23]	China	cross sectional study	200	34	166	32.28 ± 6.21	nurses	Posttraumatic Growth Inventory20 items, erceived professional benefits and intent to stay		In summary, this study found that post-traumatic growth may not promote the intent to stay
Ruey Chen, 2021 [24]	Taiwan	cross sectional study	12 596	555	12 041	33.1 ± 10.4	Nurses	Posttraumatic Growth Inventory-Short Form	≤ 10: 105(52.5), 11–20: 73(36.5), > 20: 22(11.0)	In the multiple linear regression analysis, tenure, PTGI score, emotional exhaustion, depersonalization, and lack of personal accomplishment were influential factors relating to trauma. Specifically, emotional exhaustion in the burnout dimension was the most influential factor and exhibited the highest explanatory variance
Christina Aggar, 2022 [25]	Australia	cross sectional study	767	80	678	45.93 ± 11.95	Nurses	-10-item short form of the Posttraumatic Growth Inventory, -Self-Compassion Scale–short form, -Impact of Event Scale–revised, -The Depression Anxiety Stress Scales 21, -The World Health Organisation Five Well-Being Index	21.20 ± 12.95	Posttraumatic growth reduced the negative relationship between pandemic-related stress and psychological adjustment outcomes
Yuanyuan Mo, 2022 [38]	China	cross sectional study	266	24	242	32.34 ± 6.01	Nurses	Post-traumatic Growth Inventory (PTGI), Professional Self-identity Scale, and Perceived Social Support Scale	11.35 ± 3.6	score of PTG was at a high level. There was a phenomenon of PTG when the nurses faced COVID-19 in Hubei Province. Providing an active coping style helps to improve the level of PTG
Nelson Chun-Yiu Yeung, 2022 [26]	Hong Kong	cross sectional study	1510			18–25 381 (25.2%) 26–35 555 (36.8%) 36–45 354 (23.4%) 46–55 210 (13.9%) Above 55 10 (0.7%)	nurses	Posttraumatic Growth Inventory–Short Form (PTGI-SF), -COVID-19 specific worries and current psychological distress,—Satisfaction with workplace pandemic control guidelines, -Work satisfaction, -Sociodemographic and work-related variables	8.9 ± 8.6	current distress, worries about contracting COVID-19 from the workplace, and worries about family members’ contracting COVID-19 due to their work significantly contributed to PTG among our participants
Lin Li, 2022 [27]	China	cross sectional study	455 nurses			33.51 ± 5.94	nurse and GP	- Posttraumatic growth inventory questionnaire (PTGI), -Mental health status and ways to copy with stress, -Mental health status and ways to copy with stress	16 ± 11.3	This study indicated that the score of total PTGI and three domains of PTGI, new possibilities, personal strength, and spiritual change were higher in nurses than in GP. Furthermore, sex, marriage status, professional titles, anxiety and ways to copy with stress were related to PTG in nurses
Sagit Dahan, 2022 [28]	Israel	cross sectional study	183	64	119	47.37 ± 10.71	nurses	Questionnaire PTG	Up to 5 years: 27(14.8%) 6–10: 14(7.7%) 11–15: 12( 6.6%) 16–20: 24(13.1%) 21–25: 28(15.3%) 26–30: 36( 19.7%) 30 + : 42( 23.0%)	A significant positive correlation was found between personal and national resilience (NR) and PTG. Higher professional seniority was related to higher PTG
Xin Peng, 2021 [29]	China	cross sectional study	116	10	106	34.07	nurse	post-Traumatic Growth Inventory (PTGI)	< 3: 42 3–8: 49 ≥ 9: 25	In univariable analyses, gender, age, education level, marital status, living with parents, professional title, working years and professional psychological support was not statistically associated with the PTGI score
Lulejete Prekazi, 2021 [30]	Kosovo	cross sectional study	638	408	283	41.6 ± 10.7	nurse and GP	Socio-Demographic Questionnaire, General Health Questionnaire-28, Coping Skills, Post-traumatic Growth Inventory	41.6	levels of mental health exacerbation do not play a conclusive role in determining levels of PTG, as long coping mechanisms are in place The development and implementation of interventions to minimize COVID-19-related mental health consequences, by fostering PTG among healthcare providers could be highly beneficial in pandemic response work
Hu Jiang, 2022 [31]	China	cross sectional study	3419	159	3260	30.35 ± 5.73	nuse	The General Information Questionnaire, The Questionnaire for Perceived Professional Benefits, Post-traumatic Growth Inventory, Post-traumatic Stress Disorder Scale	≤ 5 years: 2449, 6–15 years:1632, ≥ 16 years:338	The results indicated that gender, job title, department, average monthly income, the number of night shifts per month, hospital classification, specialization, and previous experience with assisting during disasters were statistically significant. The chi-square test indicated that the difference in PTSD prevalence between nurses working outside and inside Hubei Province was statistically significant and that the PTSD prevalence of nurses working outside Hubei Province was higher than that of nurses working inside Hubei Province
Ju Young Yim RN, 2022 [32]	South Korea	cross-sectional study	229	21	208	30.28 ± 4.57	nurses	Posttraumatic Growth Inventory (PTGI), Posttraumatic stress disorder (PTSD), Self-disclosure, Social support, Deliberate rumination	5.29 ± 4.01	Deliberate rumination had directly influenced posttraumatic growth and posttraumatic stress disorder and social support had a direct and indirect effect on posttraumatic growth. Self-disclosure indirectly influenced posttraumatic growth through deliberate rumination but was not significant
Arzu Sarıalioğlu, 2022 [33]	Turkey	cross sectional study	175	35	140	19–29: 83(47.4) 30–39: 35(20.0) 40 and above: 57(32.6)	Nurses	Sociodemographics Form,” “The Transformative Power of Pain Scale,” and the “Post‐Traumatic Growth Scale.”	0–2: 40(22.9) 3–10: 59(33.7) 11 and above:76(43.4)	posttraumatic growth increased as the level of the transformative power of pain increased for nurses. Furthermore, some variables were found to have an effect on the transformative power of pain and the posttraumatic growth mean score in nurses who had positive Covid‐19 PCR test
Lianrong Sun, 2022 [34]	China	cross sectional study	233	8	225	41.53 ± 6.37	nurses	Post-traumatic Growth Inventory,	4.65 ± 0.5583	Results showed that second victims (SVs) nurses’ active rumination on adverse nursing events could promote their recovery from psychological trauma, but invasive rumination could not

### Qualitative assessment

Approximately 40% and 46.6% of the studies had low and medium risk of bias, respectively. Two studies had high risk of bias. In the majority of the articles, the participation rate and sampling method were not clearly stated, making them the most notable weaknesses affecting the quality of the assessed studies (Table 3).

**Table 3:** Quality assessment of included studies in terms of risk of bias

Author, year	Was the study’s target population a close representation of the national population in relation to relevant variables, e.g. age, sex, occupation?	Was some form of random selection used to select the sample, OR, was a census undertaken( simple random sampling, stratified random sampling, cluster sampling, systematic sampling)	The response rate for the study was ≥ 75%؟	Was the study instrument that measured the parameter of interest (e.g. prevalence of low back pain) shown to have reliability and validity (if necessary)?	Risk of bias
Xin Tong Zhang, 2021 [22]	yes	not clear	not clear	yes	Moderate risk of bias
Pan pan Cui, 2020 [10]	yes	not	yes	yes	low risk of bias
Xiaoxin Liu, 2021 [23]	yes	yes	yes	yes	low risk of bias
Ruey Chen, 2020 [24]	yes	no	not clear	yes	Moderate risk of bias
Christina Aggar, 2022 [25]	somewhat	not clear	no	yes	high risk of bias
Yuanyuan Mo, 2022 [38]	yes	no	no	yes	Moderate risk of bias
Nelson Chun-Yiu Yeung, 2022 [26]	yes	no	no	yes	Moderate risk of bias
Lin Li, 2021 [27]	somewhat	not clear	not clear	yes	low risk of bias
Sagit Dahan, 2022 [28]	yes	not clear	no	yes	Moderate risk of bias
Xin Peng, 2021 [29]	yes	not clear	not clear	yes	Moderate risk of bias
Lulejete Prekazi, 2021 [30]	somewhat	not clear	not clear	yes	high risk of bias
Lianrong Sun, 2022 [34]	yes	yes	yes	yes	low risk of bias
Hu Jiang, 2022 [31]	yes	not clear	yes	yes	low risk of bias
Ju Young Yim RN, 2022 [32]	yes	no	not clear	yes	Moderate risk of bias
Arzu Sarıalioğlu, 2022 [33]	yes	yes	yes	yes	low risk of bias

### PTG prevalence

**Fig. 2:**
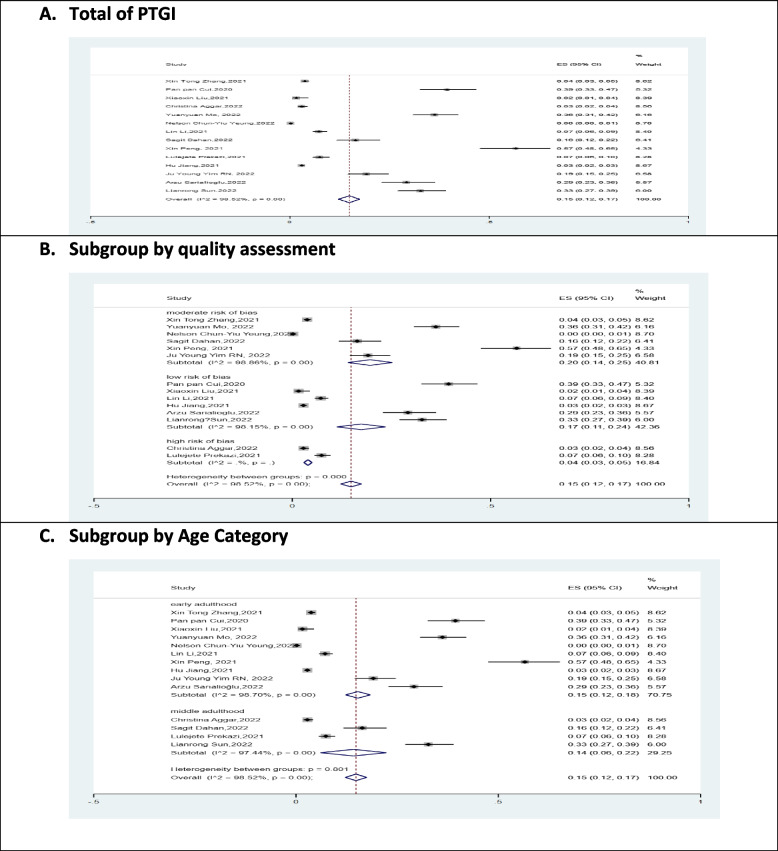
Forest plot illustrating for prevalence of PTGI in target population and sub group analysis by quality assessment of studies

The desired outcome of this study was examining the prevalence of PTG in nurses during the COVID-19 pandemic. A total of 15 studies entered the meta-analysis and PTG prevalence was randomly calculated [ES 95% Conf. Interval = 0.15[0.12–0.17]). Heterogeneity was reported I2 = 98.52% (*P* = 0.000), indicating high heterogeneity of the studies (Fig. 2). The studies were eligible for subgroup analysis to reduce heterogeneity. Thus, sub-group were analyzed in terms of types of the quality of the studies; however, severe heterogeneity was still observed (Fig. 2). Moreover, we categorized the age into two groups. Adulthood is usually classified into three phases: early adulthood or youth (approximately 20–39 years old), middle adulthood (40–59), and old age (60 +) [39]. Subgroup analysis based on age category is used to reduce heterogeneity. The Fig. 2C indicates that there is still high heterogeneity.

**Fig. 3:**
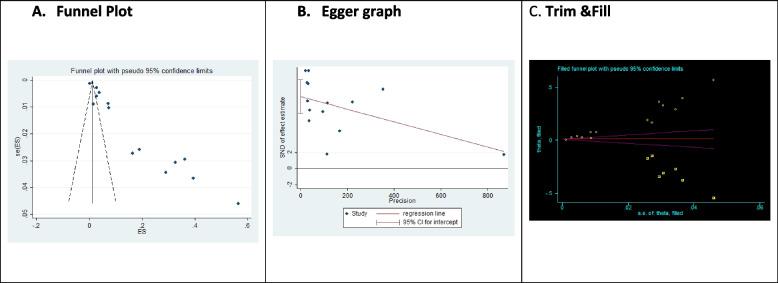
**A** funnel plots (with pseudo 95% CIs), **B** Egger graph, **C** Trim &Fill for publication bias in PTGI in population

To assess publication bias in this outcome, funnel plot, trim and fill technique, and Egger’s test were used. As demonstrated in Fig. 3, there was no symmetrical dispersion of studies, indicating publication bias. In Egger’s diagram, examining the effect of small studies, *p* = 0.000 was reported and this significant value showed the presence of publication bias. In trim and fill, additional studies up to 21 studies were recommended, and the evidence showed the presence of publication bias. Therefore, the conclusion of this study is highly affected by publication bias (*p* = 0.000).

### Meta-regression

**Fig. 4:**
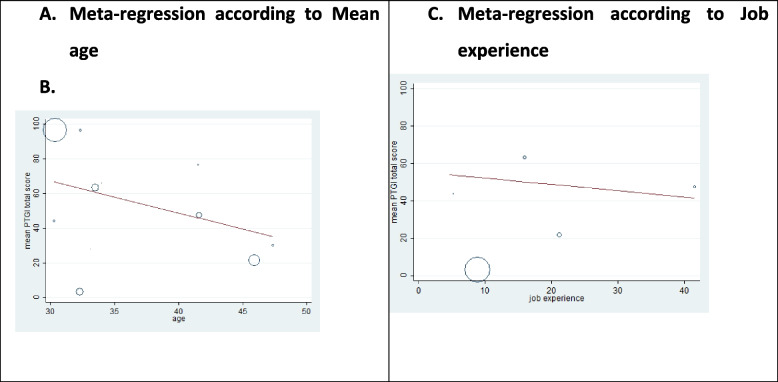
Meta-regression plot

Meta-regression, an approach suggested by Chocrane, was used for nurses’ mean age and work experience to examine the effects of potentially influential factors in PTG heterogeneity. As shown in Fig. 3, the results illustrated that with increased work experience and mean age, the mean score of PTGI decreased in nurses. However, the effect was not statistically significant for mean age (*P* = 0.06). The results indicated that the PTGI score decreased in nurses with more work experience, which was statistically significant (*P* = 0.04) (Fig. 4).

## Discussion

The recent COVID-19 outbreak and its detrimental psychological effects have brought attention to the significance of mental health for COVID-19 frontline nurses. Accordingly, this study aimed to determine the prevalence of PTG in nurses fighting against COVID-19. This meta-analysis revealed a 15 percent PTG prevalence in nurses.

Xiaoli et al. reported The level of PTG across studies ranged from 10 to 77 Percentage in their study [24]. In another study by Peng et al., COVID-19 frontline nurses had a moderate level of PTG [29]. The COVID-19 pandemic can be considered a new type of collective trauma given its particular characteristics including the global spreading of the virus, impacts on different social aspects, economic issues, advertisement and media, quarantine, and other restrictions [40]. Moreover, healthcare staff, especially nurses, suffer from additional concerns such as access to personal protective equipment, fear of infection spread, exposure to COVID-19 in the workplace, and the risk of transmitting the infection to their family members at home [5]. Although the risk of psychological problems and disorders increases in such stressful conditions, responses to crises are not necessarily negative according to the Stuart Stress Adaptation Model [5, 24]. As discussed by Tedeschi and Calhoun, some individuals reinterpret their lives through cognitive restructuring because of confronting a trauma or experiencing harm. They consciously regulate their self-perception, interpersonal relationships, and attitude to life. What happens during the growth process is the creation of a new meaning when a harmful event is processed. Managing emotional distress caused by confronting trauma and transforming intrusive rumination to deliberate rumination are influential in creating meaning and changing attitudes [35]. The results of a meta-analysis indicated that PTG had a linear and curved relationship with PTSD [41]. Nonetheless, the role of other factors such as dynamic character, social support, and self-disclosure, which are crucial in facilitating PTG, cannot be neglected [42]. Although confronting critical situations increases the risk of PTG, different demographic factors are also influential in facilitating PTG. This study showed that higher mean age and work experience can reduce the mean PTGI score in nurses. Work experience had a statistically significant relationship with PTGI score, whereas increased mean age did not. Some studies have shown that the age and work experience of nurses have no effect on PTGI score [26, 29]; however, Yeung et al. (2021) reported less PTG in full-time nurse [26]. PTG level can be influenced by factors like the effect, intensity, and importance of experienced crisis and nurses’ attitudes, cognitive structures, use of empathy, and social support [43]. The stress-inducing nature of the disease and close and continuous contact with patients are also other influential factors in increasing PTG. Experiencing constant stress, without the opportunity for cognitive-emotional restructuring, can lead to increased physical problems, job dissatisfaction, and burnout [5, 44, 45]. As a result, these factors can facilitate negative outcomes caused by fighting against the pandemic [24]. During the pandemic, which requires full-time and high-demanding work in stressful conditions, more work experience of senior nurses and probable diminishing of effective variables on growth, such as social support and sufficient time for cognitive restructuring, can be factors explaining reduced PTG in these individuals.

It should also be noted that this study had high heterogeneity. Causes of high heterogeneity in prevalence meta-analyses can be variable including differences resulting from insufficient sample size and distinct design, studied population, treatments, modifications, statistical analyses, reports, etc. [46]. High heterogeneity in this study might have been caused by difference in sample size (presence of studies with comparatively very large sample sizes), demographic differences (women to men ratio), and different reports.

In pandemics, health care workers, particularly nurses, have a vital role in screening and providing care. At the same time, stressful and hard-working conditions can have negative psychological outcomes on nurses. Therefore, adopting strategies to reduce psychological harms caused by confronting the pandemic crises and promoting growth in nurses can not only help their psychological health but also lead to providing better care to patients and others.

### Limitations

There were some limitations that should be considered in interpreting the results. Inclusion of cross-sectional studies was one major limitation. This prevented examining growth prevalence in different time periods and the effect of the duration of the pandemic on growth during the COVID-19 pandemic, also some studies had small samples. Except two studies, all examined studies had been conducted in Asian countries. Thus, the results should be generalized to nurses in other regions or countries with caution. Furthermore, a number of studies were removed because of inaccessibility of full information.

## Conclusion

This study showed a 15 percent PTG prevalence in frontline nurses during the COVID-19 pandemic. Increased years of work experience and age led to lower PTGI scores in the nurses. According to the results, psychological interventions should be planned for senior nurses with more work experience to reduce harms caused by the pandemic and crisis situation. The interventions should target job burnout and other crucial factors, aiming to train nurses to reflect on their hard work experiences purposefully and constructively and help facilitate their PTG by conversing about the importance of these experiences.

## Data Availability

The datasets used and/or analysed during the current study are available from the corresponding author on reasonable request.
